# Quincke’s Disease Presenting After Cocaine Exposure

**DOI:** 10.7759/cureus.30536

**Published:** 2022-10-21

**Authors:** Filipa Madalena F Gonçalves, Magda Costa, Ana Luísa Campos, Jorge Cotter

**Affiliations:** 1 Internal Medicine, Hospital Senhora da Oliveira Guimarães, Guimaraes, PRT; 2 Internal Medicine, Hospital Senhora da Oliveira Guimarães, Guimarães, PRT

**Keywords:** cocaine, isolated uvular angioedema, edema of the uvula, angioedematous uvula, quincke’s disease

## Abstract

Quincke’s disease is a very rare form of upper airway angioedema, and it is characterized by a well-localized edematous reaction. Its epidemiology is not documented due to the rarity of this condition. Causes include allergic reactions, infectious diseases, and trauma, among others.

This article describes a rare case of a 27-year-old man who presented to the Emergency Department with isolated uvular edema associated with cocaine exposure.

## Introduction

Angioedematous uvula results from increased vascular permeability, and usually presents as a pale, non-erythematous, and swollen uvula [1]. There might be some predisposing factors such as constitutional and mechanical conditions, for example, long uvula [2]. It was first described in 1882 by Quincke, and is therefore known as Quincke’s disease [3]. Its epidemiology is not documented due to the rarity of this condition [4,5].

Although the cause is not identified in more than half of the cases, uvular edema may be triggered by allergic reactions, infectious diseases, trauma, genetic diseases, neoplasms, or vascular alterations [1,2,6].

This article aims to describe a rare case of isolated uvular edema in the emergency department in a patient with cocaine exposure, as well as the management of a pathology of which very few reports exist in the literature. 

## Case presentation

A 27-year-old man presented to the Emergency Department (ED) with a foreign body sensation localized to the oropharynx, associated with difficulty in swallowing saliva. He denied any other symptoms, such as shortness of breath, chest pain, fever, cutaneous alterations, or pain. He had no relevant history and has not been under any chronic medication. The patient reported consuming inhaled cocaine eight hours before admission to the ED.

**Figure 1:**
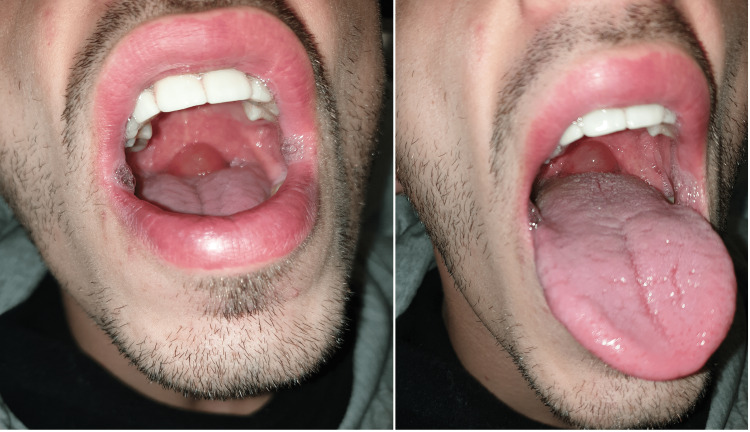
Isolated enlarged uvula

At admission, the patient was stable hemodynamically with a blood pressure of 155/81 mmHg, heart rate of 97 bpm, pulse oxymetry of 99% at fraction of inspired oxygen (FIO2) 21%, and 36.2ºC of body temperature. Physical examination showed an enlarged uvula almost 1 cm in diameter (Figure 1) with a muffled voice; without tongue or other structures edema. The cardiac auscultation was regular and no murmurs were noticed, also, pulmonary auscultation showed was symmetric, with no adventitious sounds bilaterally.

**Figure 2:**
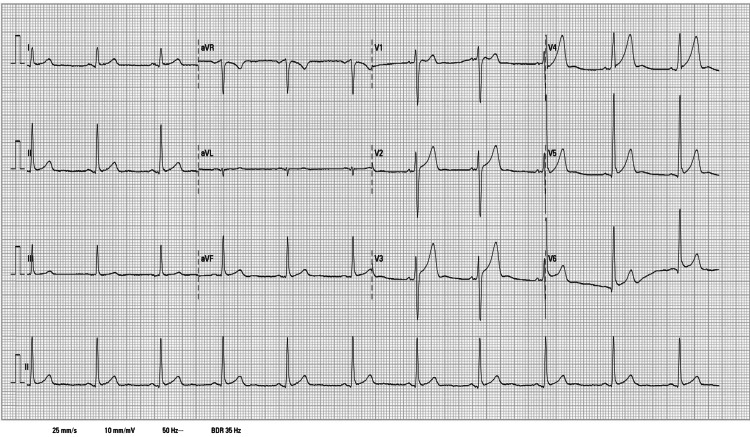
Electrocardiogram at admission

Therapy with clemastine 2 mg intravenous, hydrocortisone 400 mg intravenous, and inhaled adrenaline 2 mL was administered with rapid improvement of symptoms, allowing the patient to speak normally and establish normal swallowing. An electrocardiogram (ECG) revealed no abnormalities as shown in Figure 2.

Blood analysis is presented in Table 1, showing no significant abnormalities.

**Table 1:** Blood analysis at admission CK-MB: creatine kinase MB, pBNP: pro-brain natriuretic peptide, aPTT: activated partial thromboplastin time, INR: international normalised ratio

	Test result	Reference value
Hemoglobin (g/dL)	15.2	14.0-18.0
White blood cells (x10^3^/uL)	10.6	4.8-10.8
Neutrophils (x10^3^/uL)	8.4	1.8-7.7
Eosinophils (x10^3^/uL)	0.0	0.0-0.49
Basophils (x10^3^/uL)	0.0	0.0-0.1
Lymphocytes (x10^3^/uL)	1.7	1.0-4.8
Monocytes (x10^3^/uL)	0.4	0.12-0.80
Platelets (x10^3^/uL)	273	150-350
C-reactive protein (mg/L)	<3.0	<3.0
Urea (mg/dL)	27	15-39
Creatinine (mg/dL)	0.85	0.70-1.30
Sodium (mEq/L)	144	135-146
Potassium (mEq/L)	3.70	3.5-5.1
Aspartate aminotransferase (UI/L)	16	12-40
Alanine aminotransferase (UI/L)	17	7-40
Troponine I (ng/mL)	<0.015	<0.045
Myoglobin (ng/mL)	70	14-106
CK-MB (ng/mL)	1.01	<7.2
pBNP (pg/mL)	8.0	<125
Prothrombin Time (seg.)	11.6	8.4-14.4
aPTT (seg.)	29.5	20.9-34.9
INR	1.0	

The patient did not report any trauma or previous procedure. He maintained surveillance in the ED for 12 hours with no recurrence of symptoms or worsening of previous conditions. After that time he had no evidence of swollen uvula and no difficulty in speaking or swallowing. He was discharged asymptomatic, with a five-day steroid burst and strict return precautions. During two years of follow-up, there were no recurrence episodes.

## Discussion

Quincke’s disease is a very rare form of upper airway angioedema, and it is characterized by a well-localized edematous reaction, that may involve the deeper skin layers, subcutaneous tissues, and mucosal surfaces of the upper respiratory and gastrointestinal tract, in the absence of other constitutional symptoms [1,4,5].

It is typically caused by a Type I hypersensitivity reaction, although the exact mechanisms are not known [5]. Other causes include allergy and consumption of nonsteroidal anti-inflammatory drugs (NSAIDs), angiotensin-converting enzyme inhibitors (ACEi) and angiotensin II receptor antagonists (ARA II), and hereditary angioneurotic edema (HANE) [2,3,6]. Irritation due to inhaled substances such as cocaine and cannabis is previously described in very rare cases [2,3] and is thought to be caused due to thermal irritation, either because of the high temperature needed to burn the compounds or due to the irritation caused by the substances used to bulk out the drug [3]. It is described that a delayed presentation, when associated with cocaine, might be due to the anesthetic properties of the drug [3]. 

The clinical manifestations of acute uvular edema include a foreign body sensation muffled voice, gagging, and, if severe, signs of upper airway obstruction [1]. Other symptoms such as skin rash, hypotension, or tachycardia are not present since Quincke’s disease is not associated with other hypersensitivity reactions [5].

Laboratory workups depend on the suspected etiology, despite the ED evaluation of angioedema being limited [1]. Eosinophilia suggests an allergic reaction, and the C4 level can be used as a screening test for HANE [1].

Differential diagnoses include epiglottitis, retropharyngeal abscess, and peritonsillar abscess [4].

Treatment consists of H1 and H2 blocking drugs, steroid therapy, and, if indicated, epinephrine [5]. Due to its potent anti-inflammatory properties and long half-life, dexamethasone has been considered the corticosteroid of choice [5,7]. The main consideration is to maintain a patent airway, and, therefore, rapid access to equipment and personnel for intubation or tracheostomy is necessary [4]. When noninfectious uvulitis is present and the patient does not respond to the above measures, plasminogen inhibitor epsilonamino-caprolic acid should be considered if a complement deficiency disorder is suspected, since this agent activates C1 from its precursor stage; however, in managing acute symptoms its value is not well established [1].

Quincke’s disease has an overall good prognosis and a very low recurrence rate [5].

## Conclusions

Isolated uvular angioedema is a very rare clinical condition. There are few literature case reports. It is, as seen in our patient, a condition with a very good prognosis. Despite its potential to lead to airway obstruction, the response to medical therapy is usually efficient and results in a rapid resolution of symptoms. In this case, cocaine exposure seems to be the causative agent for this disease presentation. 
